# Critical times in pediatric out-of-hospital cardiac arrest

**DOI:** 10.1186/cc10878

**Published:** 2012-03-20

**Authors:** J Tijssen, C Zhan, C Parshuram, L Morrison, J Hutchison

**Affiliations:** 1 Hospital for Sick Children, Toronto, Canada; 2 University of Toronto, Canada

## Introduction

Pediatric out-of-hospital cardiac arrest (OHCA) has a less than 10% survival. Studies of the scene time and level of emergency medical services (EMS) training in pediatric OHCA are lacking. The objectives of this study are to describe the scene time, level of training and the order and timing of arrival of first responders to pediatric OHCA in a large, densely populated area, the Toronto region.

## Methods

The Resuscitation Outcomes Consortium (ROC) Epistry-Cardiac Arrest database was queried for all patients <19 years old from December 2005 to November 2011 in the Toronto region for age, sex, event characteristics, underlying conditions, cause of the cardiac arrest, level of EMS care, time to EMS arrival, scene time, return of spontaneous circulation (ROSC) and survival to hospital discharge. Patients were excluded if they were declared dead at the scene.

## Results

Four hundred and fifty-two patients with OHCA were included. Thirty-one percent were infants, 29.4% age 1 to 11 years (child), and 37.4% age 1 to 18 (adolescent) years with 62.8% of cases male. Thirty percent had a significant past medical history. The causes of the cardiac arrest were trauma (14.4%), drowning (6.2%), sudden infant death syndrome (4.0%), and unknown in 63%. The first EMS responders were fire in 52.2%, advanced care paramedics in 25%, and primary care paramedics in 22.3%. Survival was increased the earlier the EMS arrived (*P *= 0.015). The timing of arrival of advanced paramedics at the scene appeared to be associated with survival although this was not statistically significant (*P *= 0.22). Infants had a shorter scene time (*P *< 0.001) and an earlier arrival of advanced care paramedics at the scene (*P *= 0.04). A shorter scene time was associated with ROSC on arrival at the emergency department (*P *< 0.001) and a nonsignificant trend for improved survival (*P *= 0.13). Adolescents were more likely to have ROSC on arrival at the emergency department (*P *< 0.001) and more likely to survive (*P *< 0.05) compared to children or infants.

## Conclusion

The timing of arrival of advanced paramedics at the scene may have been associated with survival and a larger study is needed to confirm this trend. A shorter scene time was associated with ROSC and a trend for increased survival. However, infants have shorter scene times but worse outcomes. To provide increased power and scope for this study we will expand it to include all 10 Regional Clinical ROC Centers and future analyses will include the remaining Utstein data fields and compare the effects of advanced versus basic life support interventions during resuscitation.

